# Supplementary material for: The rule-based insensitivity effect: a systematic review
Source: PeerJ. 2020 Jul 23;8:e9496. doi: 10.7717/peerj.9496 (PMC7382939; doi:10.7717/peerj.9496)
Supplement: Supplemental Information 1 [file peerj-08-9496-s001.docx]

**PRISMA CHECKLIST**

| **Section/topic** | **#** | **Checklist item** | **Reported on page #** |
| --- | --- | --- | --- |
| **TITLE** | | |  |
| Title | 1 | Identify the report as a systematic review, meta-analysis, or both. | Page 1  (The rule-based insensitivity effect: a systematic review) |
| **ABSTRACT** | | |  |
| Structured summary | 2 | Provide a structured summary including, as applicable: background; objectives; data sources; study eligibility criteria, participants, and interventions; study appraisal and synthesis methods; results; limitations; conclusions and implications of key findings; systematic review registration number. | Page 2  **(Background.** Adherence to inaccurate rules has been viewed as a characteristic of human rule-following (i.e., the rule-based insensitivity effect; RBIE) and has been thought to be exacerbated in individuals suffering from clinical conditions. This review intended to systematically examine these claims in adult populations.  **Methodology.** We screened 1464 records which resulted in 21 studies that were deemed eligible for inclusion. Each of these studies was examined to determine: (1) if there is evidence for the RBIE in adults and (2) if this effect is larger in those suffering from psychological problems compared to their non-suffering counterparts. In addition, we investigated how (3) different operationalizations of the RBIE, and (4) the external validity and risks of bias of the experimental work investigating this effect, might influence the conclusions that can be drawn from the current systematic review.  **Results.** (1) Out of the 20 studies that were relevant for examining if evidence exists for the RBIE in adults, only 11 were eligible for vote counting. Results showed that after the contingency change, the rule groups were more inclined to demonstrate behavior that was reinforced before the change, compared to their non-instructed counterparts. Critically, however, none of these studies examined if their no-instructions group was an adequate comparison group. As a result, this made it difficult to determine whether the effects that were observed in the rules groups could be attributed to the rules or instructions that were manipulated in those experiments. (2) The single study that was relevant for examining if adults suffering from psychological problems demonstrated larger levels of the RBIE, compared to their non-clinical counterparts, was not eligible for vote counting. As a result, no conclusions could be drawn about the extent to which psychological problems moderated the RBIE in that study. 3) Similar procedures and tasks have been used to examine the RBIE, but their precise parameters differ across studies; and (4) most studies report insufficient information to evaluate all relevant aspects affecting their external validity and risks of bias.  **Conclusions.** Despite the widespread appeal that the RBIE has enjoyed, this systematic review indicates that, at present, only preliminary evidence exists for the idea that adults demonstrate the RBIE and no evidence is available to assume that psychological problems exacerbate the RBIE in adults. ) |
| **INTRODUCTION** | | |  |
| Rationale | 3 | Describe the rationale for the review in the context of what is already known. | Pages 3-4  (Over the past decades, a number of studies have empirically examined the RBIE in the laboratory (e.g., Donadeli & Strapasson, 2015; Joyce & Chase, 1990; Miller, Hirst, Kaplan, DiGennaro Reed, & Reed, 2014; Ninness & Ninness, 1998). Elsewhere, applied researchers and clinical psychologists have appealed to this effect when attempting to understand and treat psychological suffering. For instance, it has been argued that the RBIE is at the core of various problems such as addiction, depression, and personality disorders (Baruch, Kanter, Busch, Richardson, & Barnes-Holmes, 2007; Blackledge & Drake, 2013; Hayes & Gifford, 1997; McAuliffe, Hughes, & Barnes-Holmes, 2014; Törneke, Luciano, & Salas, 2008; Törneke, 2010). The idea here is that psychological problems are – amongst other things – the consequence of adherence to rules that reduce one’s ability to persist or adapt to what is required in a given situation (Blackledge & Drake, 2013).  Nevertheless, and despite the attention that rules and the RBIE have received, there is currently no systematic review available of the experimental work examining this effect. This is unfortunate, given that such a review is essential to draw general conclusions about the RBIE which can inform future research and clinical practice. Towards this end, we systematically reviewed the RBIE literature to examine if: (1) there is sufficient empirical support for this effect in adults, and (2) adults suffering from psychological problems display larger levels of this effect compared to those that do not suffer from these problems. We also investigated (3) how different operationalizations of the RBIE, and (4) the external validity and risks of bias of the experimental work investigating this effect, might influence the conclusions that can be drawn from the current systematic review.) |
| Objectives | 4 | Provide an explicit statement of questions being addressed with reference to participants, interventions, comparisons, outcomes, and study design (PICOS). | Page 4  (… we systematically reviewed the RBIE literature to examine if: 1) there is sufficient empirical support for this effect in adults, and (2) adults suffering from psychological problems display larger levels of this effect compared to those that do not suffer from these problems. In addition, we investigated (3) how different operationalizations of the RBIE, and (4) the external validity and risks of bias of the experimental work investigating this effect, might influence the conclusions that can be drawn from the current systematic review.) |
| **METHODS** | | |  |
| Protocol and registration | 5 | Indicate if a review protocol exists, if and where it can be accessed (e.g., Web address), and, if available, provide registration information including registration number. | Page 4  (The review protocol was designed in line with the PRISMA guidelines (Moher, Liberati, Tetzlaff, & Altman, 2009) and registered in PROSPERO (CRD42018088210).) |
| Eligibility criteria | 6 | Specify study characteristics (e.g., PICOS, length of follow-up) and report characteristics (e.g., years considered, language, publication status) used as criteria for eligibility, giving rationale. | Pages 4-5  (There were several general criteria that a record had to meet before being included in the current review: (1) it had to be a peer-reviewed journal article, (2) it had to be written in English, (3) it had to include a study that examined the RBIE by first asking participants to follow socially –or self-generated rules that initially corresponded with a set of contingencies but then became inaccurate after a contingency change, and (4) this study had to have an overall sample age of at least 18 years, (5) and at least 10 participants within each experimental group (see Van Ryckeghem, Van Damme, Eccleston, & Crombez, 2018 for similar eligibility criteria).  Furthermore, depending on the research objective under scrutiny, the individual studies reported in these records had to meet an additional number of criteria to be deemed eligible for inclusion. For instance, when addressing our first research question (“*Is there evidence for the rule-based insensitivity effect in adults?”*), we only included studies that did not focus upon individuals with clinical problems. That is, only studies which used convenience samples (e.g., students), samples taken from the general population, or those that were not diagnosed with clinical problems, or reported sub-clinical problems were included. Studies were deemed eligible for answering our second research question (*“Do adults suffering from psychological problems display a larger RBIE compared to their non-clinical counterparts?”*), if they used the following samples: individuals diagnosed with psychological problems (clinical group) or those who scored high on instruments measuring psychological problems but were not formally diagnosed with a clinical problem (sub-clinical group), *and* a comparison group consisting of individuals that did not suffer from the above problems or were recruited via convenience sampling.) |
| Information sources | 7 | Describe all information sources (e.g., databases with dates of coverage, contact with study authors to identify additional studies) in the search and date last searched. | Page 4  (To identify as many relevant records as possible, multiple electronic databases were searched (i.e., “Web of Science”, “PsychINFO”, “PsychArticles”, and “PubMed [Medline]”)  …  All searches were conducted on 4/10/2017 by the first author (i.e., Ama Kissi) and yielded 1459 records. Five novel records were additionally retrieved by contacting experts in the field, which resulted in a final set of 1464 records that were assessed for eligibility.) |
| Search | 8 | Present full electronic search strategy for at least one database, including any limits used, such that it could be repeated. | Page 4  (“rule governed behavior”, “rule-governed behavior”, “rule governed behaviour”, “rule-governed behaviour”, “verbal regulation”, “instructional control”, “verbal rule”, “instructed behavior”, “instructed behaviour”, “instructed learning”, “instruction following”, “instruction-following”, “rule following”, and “rule-following.”) |
| Study selection | 9 | State the process for selecting studies (i.e., screening, eligibility, included in systematic review, and, if applicable, included in the meta-analysis). | Page 5  (Out of the 1464 records that were assessed for eligibility, 1446 were excluded because they were not published in English (*n* = 123), were not peer-reviewed journal articles (*n* = 207) (e.g., book chapters, dissertations, or conference papers) or dealt with a topic that did not meet our inclusion criteria (*n* = 1044). Three journal articles were, furthermore, omitted because they did not provide sufficient information to assess their eligibility. An additional 69 journal articles were excluded that were on the RBIE but were non-experimental (*n* = 6), relied on non-adult samples (*n* = 14), used samples with less than 10 participants per experimental condition (*n* = 41), or did not include a contingency change or manipulate accurate rules (*n* = 8). This resulted in a remaining total of 18 records consisting of 22 individual studies. One of these studies was subsequently omitted because it did not have at least 10 participants within each experimental group. As such, 21 studies were finally included in the systematic review. The eligibility of all studies were independently assessed by the first two reviewers (i.e., Ama Kissi and Colin Harte) initial agreement = 99% [kappa = .98], agreement after discussion = 100% [kappa = 1.00]).) |
| Data collection process | 10 | Describe method of data extraction from reports (e.g., piloted forms, independently, in duplicate) and any processes for obtaining and confirming data from investigators. | Pages 5  (Certain characteristics of each of the 21 studies were independently coded by the first two reviewers (i.e., Ama Kissi and Colin Harte) (initial inter-reviewer agreement = 96%, inter-reviewer agreement after discussion = 100%).) |
| Data items | 11 | List and define all variables for which data were sought (e.g., PICOS, funding sources) and any assumptions and simplifications made. | Pages 5-6  (These characteristics involved the source, study, task, and sample characteristics. The source characteristics entailed the year in which the first author published the study and the country where s/he worked in when the paper was published. The study characteristics referred to the type of task, experimental design, procedure, and analytic method that were used to examine the RBIE. Furthermore, the task characteristics entailed whether a study reported the exact instructions or rules that were used, how these instructions or rules were delivered (orally versus written) or generated (self [i.e., by the rule-follower]-versus socially [i.e., by another person than the rule-follower]), the reinforcement schedules that were used, the required behavioral responses, the type of consequential stimuli that were used, whether the contingency change was (un)signaled, whether a description was provided of who the experimenter was, and whether the experimenter was present. Finally, the sample characteristics that were evaluated were the size and mean age of the sample, the ratio of males:females, and whether the sample was selected (i.e., from either a healthy, clinical or sub-clinical population, or the general population) or non-selected (i.e., a convenience sample). These characteristics were evaluated for each experimental group).) |
| Risk of bias in individual studies | 12 | Describe methods used for assessing risk of bias of individual studies (including specification of whether this was done at the study or outcome level), and how this information is to be used in any data synthesis. | Pages 7-8  (We, additionally, scrutinized the internal validity of the included studies. This examination involved assessing risks of bias using the Cochrane Collaboration tool for assessing risks of bias (Higgins & Altman, 2008) and the Office of Health Assessment and Translation (OHAT) Risk of Bias Rating Tool (NTP, 2015). Risks of bias can be defined as those aspects of a study design that can distort the conclusions that can be drawn from it. For the present review, we evaluated five potential risks of bias: *selection, exclusion, performance, detection, and reporting bias*. Note that these biases do not cover all risks of bias that are described in the Cochrane Collaboration and OHAT risks of bias tools. Indeed, given that these tools were not originally developed for assessing risks of bias in experimental-behavioral research, we selected and reformulated those risks of bias that we deemed relevant for evaluating such work.  For each of the studies, judgments of risks of bias (coded in terms of ‘high’, ‘low’, or ‘unclear’ risk of bias) were made in the following ways. To examine the possibility that there were systematic differences between the baseline characteristics of the groups that were compared (i.e., a *selection bias*), we examined: 1) the adequateness of a study’s sequence generation procedure, 2) whether the experimental group to which participants were allocated to was concealed, 3) participants’ past experiences with the experiment, and 4) the possibility that they were misclassified to experimental groups. Furthermore, to assess the likelihood of an *exclusion bias* (i.e., systematic differences in the exclusion of participants from a study) we evaluated the possibility that there were systematic differences between groups with regard to the amount, nature, and handling of missing outcome data. To determine the risk of a *detection bias* (i.e., systematic differences between groups in how outcomes are determined) we evaluated: 1) the validity and reliability of the outcome assessment methods, 2) the adequateness of the outcome assessments, 3) the adequateness of the methods that were used to determine sample sizes and 4) the adequateness of the methods used to analyze the results. Judgments concerning *performance biases* (i.e., systematic differences between groups in how they were treated or exposed to factors other than the manipulation of interest) were made by examining whether: 1) the experimental contexts were standardized, 2) participants were informed about the study objectives, and 3) researchers and/or participants were informed about the experimental group to which participants were allocated to. Finally, to assess the possibility of a *reporting bias* (i.e., systematic differences between reported and unreported findings) we assessed potential discrepancies between the outcomes that were specified prior to the study and those that were eventually reported.) |
| Summary measures | 13 | State the principal summary measures (e.g., risk ratio, difference in means). | Pages 6-7  (The outcome data that were preferably used to cast votes were measures of the central tendency (e.g., mean, mode, or median) of participants’ responses, during all blocks after the contingency change. If a study, however, did not report participants’ performances during all blocks following the contingency change, but only during a fraction of the trials after this change, we limited our analysis to that data. In the unfortunate event that no data was provided that could be used to draw conclusions about the central tendency of participants’ responding after the contingency change, we relied on the conclusions that the authors formulated themselves (Cerutti, 1991; Torgrud, Holborn, & Zak, 2006 [Experiments 1 and 2]). Finally, in all of the above cases, if there were multiple contingency changes we only considered participants’ responding after the *first* change. This was, specifically, done to prevent carry-over effects from influencing the interpretation of the results.) |
| Synthesis of results | 14 | Describe the methods of handling data and combining results of studies, if done, including measures of consistency (e.g., I^2^) for each meta-analysis. | Pages 6-7  (To synthesize the quantitative results of the included studies, we used the vote-counting method. This method was chosen because not all studies reported effect sizes or information that could be used to calculate such estimates. According to the Cochrane Collaboration guidelines for systematic reviews, the best way to use the vote-counting method is by assessing whether the results of the empirical studies fall into one of two categories: “*positive*” or “*negative*” effects (see Deeks, Higgins & Altman, 2008). *Positive* effects refer to results that are in favor of the predicted relationship between the independent and dependent variable(s), whereas *negative* effects refer to outcomes that are in the opposite direction of what is expected. We only judged (or voted) whether a study had positive or negative effects if it included a comparison group (i.e., a no-instructions group). That is, a group that received the same treatment as the rules group but was not asked to follow the instructions or rules that these groups had to follow. We applied this restriction because we argued that such a comparison group is necessary if a study wishes to draw conclusions about the extent to which certain rules or instructions are responsible for the observed effects. In doing so, performances in the comparison group would serve as a baseline of how people behave in the absence of these types of rules or instructions. As such, if a study did not include such a comparison group, we argued that its effects were *unclear* (i.e., there was insufficient information to cast votes).  …  All votes were independently cast by the first two reviewers (i.e., Ama Kissi and Colin Harte) in the following manner (inter-reviewer agreement = 100%, kappa = 1.00). For the first research question (“*Is there evidence for the rule-based insensitivity effect in adults”*), study results were considered *positive* if evidence was found for the RBIE. That is, if participants did not adapt to a novel task-contingency or rule (i.e., if their behavior was still in line with the self-generated or socially-provided rule that was in place before the contingency change). Furthermore, study results were considered *negative* if one of three conditions were met. First, if a task-contingency was changed and participants’ behavior was now always in line with this novel contingency. Second, if a self-generated or socially-provided rule was altered, and participants’ behavior was now always in accordance with this novel rule. Third, if both a task-contingency *and* rule was changed, and participants’ behavior was now always in line with this novel contingency *and* rule.  To cast votes for the second research question (“*Do adults suffering from psychological problems display a larger RBIE compared to their non-clinical counterparts?*”), we first assessed whether there was evidence supporting the RBIE. This was achieved in the same way as outlined above. If evidence for the effect was found, we subsequently examined if it was larger (in absolute terms) in the (sub-)clinical groups, compared to their non-clinical counterparts. If this was the case, then the study results would be categorized as *positive*. If these results were in the opposite direction, we would categorize them as *negative*.) |
