# Supplementary material for: The rule-based insensitivity effect: a systematic review
Source: PeerJ. 2020 Jul 23;8:e9496. doi: 10.7717/peerj.9496 (PMC7382939; doi:10.7717/peerj.9496)
Supplement: Supplemental Information 2 [file peerj-08-9496-s002.docx]

**Studies included to answer Research Question 1.** Cerutti (1991) examined the moderating effects of mixed-random, mixed-fixed, and fixed-time schedules on the manner in which participants adapted to changes in the reinforcement delivered for self-generated rules about the task-contingencies. Participants were presented with one of three schedules (a mixed-random time schedule [*n* = 10]; a mixed-fixed time schedule [*n* = 11]; a fixed-time (FT) 3.3 schedule [*n*= 10]). During each of these schedules they were asked to avoid the occurrence of tones by pressing one of two panels (A & B), and to earn points by guessing how they could prevent these tones (i.e., by generating rules about these contingencies). During the initial phase of the experiment, participants earned points if they indicated that they thought that pressing panel A rapidly prevented the tones. In the second phase, however, these contingencies changed so that now points were only earned for high-rate guesses for panel B. Note, that these points for guesses were not contingent upon the extent to which they accurately reflected the task-contingencies, but were randomly shaped. The results indicated that participants in the mixed schedules groups were inclined to demonstrate behavior that was in line with what they thought prevented the tones (e.g., pressing fast or slow), despite the non-corresponding contingencies, while this was not the case in the FT schedule group.

Once again in 1994, Cerutti investigated the effects of three different types of reinforcements schedules on participants’ adaptation to changes in self-generated rules using a similar paradigm as in his 1991 study. The most essential procedural difference between both studies was that now participants were quasi-randomly assigned to either a random-interval (RI) 10 schedule (*n* = 20), fixed-interval (FI) 10 schedule (*n* = 20), or FI 10 schedule with videotaping (*n* = 20). The results suggested that when the reinforcement contingencies for the guesses (i.e., self-generated rules) were reversed (i.e., when high-rate guesses for panel B instead of panel A were reinforced), compliance with these reversed guesses was more likely under the FI schedule with videotaped performance and the RI schedule, compared to the FI schedule alone. The RI and FI schedule with videotaped performance, however, did not differ in the extent to which they adhered to the reversed guesses.

Dixon, Hayes, and Aban (2000) examined the effects of the accuracy of instructions on behavior, when the chances of receiving reinforcement were rendered low. Participants randomly received accurate (*n* = 15), inaccurate (*n* = 15) or no-instructions (*n* = 15) about how they should play a game of roulette. When these instructions were presented this was accompanied by payback percentages of *p* = .2, *p* = .8 or those that were fair. In the next phase of the experiment, the winning probabilities were all set to *p* = .2 and participants were given the opportunity to quit the game. Results showed that participants who received instructions were more likely to quit the game compared to those that were not given any instructions. This tendency was, furthermore, larger in the inaccurate compared to accurate instructions group, indicating that the former group behaved less in line with the new reinforcement schedule compared to the latter group. No comparison could be made between the different winning probability groups, given that *N* < 10 within each of these groups.

Haas and Hayes (2006) examined the unique and combinatory effects of two types of verbal feedback: rule-following and task performance feedback, and the accuracy of rule-following feedback on participants’ adaptation to changes in the task-contingencies. Participants were randomly allocated to one of six groups (10 in each group): the inaccurate rule-following feedback, accurate rule-following feedback, inaccurate rule-following + task performance feedback, accurate rule-following + task performance feedback, rule alone or minimal rule group. In each of these groups, participants had to move a shape on a screen through a grid to earn points. Before starting the task, all participants, except those in the minimal rule group, received accurate instructions about how they could earn points during Phase 1. Specifically, these participants were told that points could be earned by pressing the buttons slowly if the blue rectangle is lit and rapidly when the red rectangle is lit (both of which appeared on the screen below the grid). The reinforcement schedules that were in effect during Phase 1 were a Differential Reinforcement of Low rates (DRL) 6 schedule when the blue rectangle was lit and a Fixed-Ratio (FR) 18 schedule when the red rectangle was lit. Towards the end of Phase 1, participants received feedback about whether their behavior corresponded with the rules they received and/or their task performances (depending on their experimental group). This feedback was either accurate (in the accurate rule groups) or inaccurate (i.e., non-contingently positive in the inaccurate rule groups). During Phase 2, the task-contingencies changed so that now reinforcement was delivered according to a multiple FR 1 schedule when the blue rectangle was lit, and an FI yoked schedule (i.e., the interval reflected the average number of seconds that participants needed to respond 18 times during the last FR component) when the red rectangle appeared. The results indicated that, on average, participants failed to adapt to the changes in the reinforcement schedules fully. This was mainly the case in the accurate rule-following with task performance feedback group when the DRL 6 schedule changed to an FR 1 schedule, and the accurate rule-following feedback without task performance feedback group when the FR 18 schedule changed to an FI yoked schedule.

Across two experiments Harte, Barnes-Holmes, Barnes-Holmes, and McEnteggart (2017) examined how receiving a direct rule versus deriving a rule affected how participants adapted to changes in reinforcement contingencies. In Experiment 1, participants were randomly assigned to either a direct (*n* = 25) or a derived (*n* = 44) rule group. In Phase 1, participants completed a conditional discrimination task in which they initially always received points if they correctly matched stimuli according to their physical dissimilarities. In Phase 2, however, the task-contingencies were reversed so that now points could only be earned if participants correctly matched stimuli according to their physical similarities. The results showed that, of those participants that met the specific performance criteria, after the contingency reversal, both the direct and derived rule groups adhered to the rules that were effective prior to the reversal. This effect, however, appeared to be slightly larger in the direct compared to the derived rule group.

In Experiment 2, Harte and colleagues tried replicating this finding using a similar procedure as in Experiment 1, with two notable exceptions. First, participants now had more opportunities to follow the reinforced rule in Phase 1 than in Experiment 1 (10 trials in Exp. 1 vs. 100 in Exp. 2). Second, a comparison group was also included that did not receive rules about how to earn points, and as such had to detect the task-contingencies themselves. Twenty-five participants were assigned to this group, while the remaining participants were randomly allocated to the direct (*n* = 39) or derived (*n* = 76) rule groups. The results suggested that, of those participants that met the specific performance criteria, all groups were somehow inclined to demonstrate behavior that was reinforced before the contingency reversal. This tendency, however, appeared to be the largest in the direct rule group, followed by the derived rule group and then the comparison group.

Hayes et al. (1986) examined whether initially partially accurate (*n* = 13), accurate (*n* = 16) or no-instructions (*n* = 19) regarding appropriate rates of responding, influenced participants behavior during extinction. Irrespective of the instructions that were given, all participants could initially earn points if they pressed buttons according to a DRL 6 schedule when a yellow rectangle was lit, and FR 18 schedule when a blue square was lit. After a certain period, an extinction phase was introduced during which responses were no longer reinforced. The results showed that, on average, almost all participants continued to emit responses during extinction (i.e., after the task-contingency change). This was more so for the accurate instructions group compared to the partially accurate and no-instructions groups, and for the no-instructions group compared to the partially accurate instructions group.

Kissi et al. (2018) examined the moderating effects of two types of rules (plys and tracks ^^[[1]](#footnote-1)^^) on participants’ adaptation to a task-contingency change. Participants were randomly assigned to one of three groups: a ply (*n* = 15), track (*n* = 17) or no-instructions (*n* = 13) group. In each group, participants had to complete a conditional discrimination task consisting of two phases. During Phase 1, they always received points for matching stimuli according to their physical similarities, while during Phase 2 points were always delivered for matching stimuli according to their physical dissimilarities. Before completing both phases, participants in the rules groups received accurate instructions about the task-contingencies of Phase 1. The no-instructions group, however, did not receive such information and as such had to learn about these contingencies via trial-and-error. The results, of the data of those participants that were included for analyses, showed that when the contingencies reversed (Phase 2), participants were generally inclined to stick to behavior that was reinforced during Phase 1. This was more so for the instruction groups compared to the no-instructions group, and for the ply compared to the track group.

Kudadjie-Gyamfi & Rachlin (2002) examined the impact of rule-governed versus contingency shaped behavior on adaption to task-contingency changes. Eighty participants were randomly divided into an instruction (*n* = 40) and a no-instruction (*n* = 40) group. In each group, participants had to press one of two buttons (Button 1 or 2) in order to earn points and minimize the delays between consecutive trials. During Phase 1 of the task, pressing Button 2 rather than Button 1 was more effective, because this maximized point earnings while reducing the delays between consecutive trials. During Phase 2, however, these contingencies were reversed so that now pressing Button 2 rather than Button 1 was more advantageous (in terms of more points and smaller time-delays). Before beginning the task, participants in the instructions group received accurate instructions about the task-contingencies during Phase 1, while no such information was provided to the no-instructions group. Results suggested that during Phase 2, all groups were likely to continue selecting Button 1, but this tendency was higher in the instructions groups compared to the no-instructions group.

Lefrancois, Chase, and Joyce (1988) examined how receiving accurate instructions or no instructions about how to earn points differentially affected participants’ adaptation to changes in reinforcement schedules. Participants were randomly assigned to one of six groups: Variety 1 instructions multiple reinforcement schedules (*n* = 15), Variety 2 instructions multiple reinforcement schedules (*n* = 15), Specific instructions Variable-Interval (VI) schedule (*n* = 15), Specific instructions Variable-Ratio (VR) schedule (*n* = 15), Minimal instructions VI schedule (*n* = 15) or Minimal instructions VR schedule (*n* = 15) group. During Phase 1 of the task, all groups except the Minimal instruction groups, received instructions which accurately described the way to earn points. In the variety instructions groups, multiple accurate instructions were given across a variety of reinforcement schedules, while in the specific instructions groups only one such instruction was provided under a single reinforcement schedule. During Phase 2, the task-contingencies were changed so that participants now had to earn points under an FI 30 schedule. The results showed that all groups did not behave in line with the novel reinforcement schedule. In fact, the Minimal instructions groups and the Specific instruction VR schedule group deviated the most from the task-contingencies (i.e., emitted more responses during the FI 30 schedule) compared to the Specific instruction VI schedule and the Variety instructions groups.

Monestès et al. (2017) examined whether rule-based insensitivity to task-contingency changes would generalize to other indirectly related and novel task-contingencies. In this study, participants were required to complete two tasks. In Task 1, they had to earn as many points as possible according to a VR 8 and a DRL 8 schedule in the presence of nonsense words A and B, respectively. During Task 2, participants had to match nonsense words according to the equivalence class in which they were being trained. Depending on the condition to which they were allocated, participants either received (*n* = 46) or did not receive (*n* = 41) any instructions about the task-contingencies in both tasks. Following completion of Tasks 1 and 2, participants were required to complete Task 3. This was largely similar to the first task, with two exceptions. First, instead of using the nonsense words A and B, other nonsense words that were in the same equivalence classes as these words (trained in Task 2) were used. Second, the reinforcement contingencies were now reversed so that reinforcement was delivered according to a VR 8 schedule when stimuli in the same equivalence class as nonsense word B were shown, and a DRL 8 schedule when those belonging to equivalence class A were presented. The results, of the data of those participants that were included for analyses, revealed that both the instructions and no-instructions groups failed to fully adapt to the reversed task-contingencies during Task 3. However, this tendency was greater in the instructions compared to the no-instructions group.

Monestès et al. (2014) examined the impact of different types of instructions or no-instruction upon participants’ reactions to changes in task-contingencies. Participants were either randomly provided with socially-generated instructions about the task-contingencies (*n* = 10), asked to generated their own rules about these contingencies (*n* = 10) or not giving any instructions about how they should respond in the task (*n* = 10). Next, they completed a task in which points could be initially earned for pressing a right button according to an FR 8 schedule, and a left one according to an FI 8 schedule. After a while, the initial task-contingencies were reversed such that points were now delivered according to an FR 8 schedule for left button presses and an FI 8 schedule for right button presses. The results showed that when the task-contingencies reversed, participants failed to adapt to this reversal (i.e., they continued to press the right button more frequently than the left button). This was more the case in the socially-provided rule group, followed by the self-instructed group, and then the no-instructions group.

In two experiments (Experiment 1: *n* = 100; Experiment 2: *n* = 96), Otto, Torgrud, and Holborn (1999) tested the effects of instructions on participants’ adaptation to contradicting task-contingencies. Participants were required to press computer keys to move a cursor through a matrix. Points for cursor movements were initially delivered under a multiple FR 18 and a DRL 6 schedule, where each component alternated every few minutes. Before being exposed to this phase, participants received accurate instructions to go fast and slow when the FR 18 and DRL 6 schedules were in effect, respectively. After a while, the task-contingencies were reversed so that now participants were instructed to go fast when the DRL 6 and slow when the FR 18 schedules were in effect. The results showed that, in both Experiments, participants failed to adapt fully to the task-contingency change.

Shimoff, Catania, and Matthews (1981; Experiment 1) examined how instructed versus non-instructed participants adapted to task-contingency changes. In this study, participants could initially earn points by pressing a button slowly during a combined Random-Interval (RI) 15 and DRL 3 schedule. After a while, however, the reinforcement contingency during the DRL 3 schedule was removed, so that points could only be earned under the RI 15 schedule. Before initiating the experimental task, participants were either accurately informed about the task-contingencies that were in effect prior to the contingency change (but not those after this change) (*n* = 10) or received no such information (*n* = 11). Results showed that after the contingency change, both groups failed to behave in line with this change and that this effect was larger for participants that were given instructions.

Souza, Pontes, and Abreu-Rodrigues (2012) investigated the effects of changes in the accuracy of instructions to emit systematic or random digit sequences on participants’ behavior. To evaluate this, Souza et al. randomly assigned participants to a systematic (*n* = 12) or random instructions (*n* = 12) group, or a group that did not receive instructions about the task-contingencies (*n* = 12). In each of these groups, participants completed a task in which they had to type sequences of three digits which, if correct, were always rewarded with points. During the first phase of the task, a sequence was considered correct if it a) differed from the two previous sequences and b) had a weighted relative frequency that was less than or equal to a certain threshold. During the second phase, however, this contingency was omitted and, as a result, responding no longer produced reinforcement. Results, of the data of those participants that were included for analyses, indicated that during the second phase, all participants continued to respond in ways that were effective during Phase 1. This effect was slightly more pronounced in the systematic instructions group compared to the random and no-instructions groups.

Svartdal (1989), examined how receiving instructions affected adjustment to inaccurate response-feedback. Participants completed a task in which they were told to count and correctly report the number of auditory stimuli they heard. During the first few trials, no feedback was provided about the accuracy of their reports (i.e., baseline). After a while, however, participants received feedback about their reports (i.e., during the feedback trials). Unbeknownst to participants, this feedback was not based on the accuracy of their reports but rather on the rate with which they reported the number of stimuli they heard. That is, feedback was delivered whenever participants’ mean rate of responding was either below (Slow group; *n*=14) or above (Fast group; *n* =13) their baseline rate of responding. According to the authors, during the feedback trials, participants in the Slow group were slower and those in the Fast group faster to emit responses (compared to baseline), which suggests that participants generally adapted to the novel contingencies. This tendency, however, was slightly more pronounced in the Slow compared to the Fast group.

Svartdal (1995; Experiment 2) explored the impact of instructions on participants’ adaption to changes in both instructed- and task-contingencies. First, participants were informed that during the first part of the task, correct responding would be reinforced with a light signal whenever they pressed a key once every second. They were then told that during Part 2, reinforcement (i.e., a light signal) would be delivered if they slightly decreased (*n* = 12; Decrease group)^^[[2]](#footnote-2)^^ or increased (*n* = 12; Increase group)^^[[3]](#footnote-3)^^ their response rate and kept this rate as stable as possible for the remainder of the experiment. Participants were additionally informed that during Part 2, feedback about their responding would be less informative and that they should, therefore, base their performances on what they had learned from Phase 1. Results showed that participants adapted to the contingency change, given that during Phase 2 rates of responding declined and augmented in the Decrease and Increase groups, respectively.

Torgrud et al. (2006; Experiment 1) examined how initially accurate instructions on either a functional or non-functional multiple reinforcement schedule, or a VR 8 schedule differently impacted participant responding on an FI 30 schedule. All participants were randomly assigned to one of three groups: the functional multiple (*n* = 15), non-functional multiple (*n* = 15) or single (*n* = 15) schedule group. In each of these groups, participants were instructed to try to earn as many points as possible in order to increase their chances of winning a monetary reward. In the multiple schedule groups, participants initially received instructions which accurately informed them about how they could earn points by pressing an “earn” key under an FR, a DRL, and a VI schedule. These contingencies could either be functional or non-functional depending on whether they trained a response rate that was or was not beneficial under the FI 30 schedule, respectively. Participants in the single schedule group, however, only received accurate instructions which initially informed them about how they could earn points under a VR 8 schedule. After some trials, all participants were then exposed to the FI 30 schedule. The findings showed that all groups failed to adapt to the last two minutes of this schedule. This was more pronounced in the single schedule group compared to the other groups, and in the non-functional schedule group compared to the functional schedule group.

Torgrud et al. (2006) attempted to replicate and extend these findings in a second experiment in which 150 participants were randomly assigned to one of six multiple schedule groups: Functional FR (F-FR), Non-Functional FR (NF-FR), Functional DRL (F-DRL), Non-Functional DRL (NF-DRL), Functional FR and DRL (F-BOTH) or Non-Functional FR and DRL (NF-BOTH), or a single schedule group. As in the previous experiment, participants initially received accurate instructions before being exposed to an FI schedule (now an FI 15 as opposed to an FI 30). This time, these instructions described how participants could earn points during an FR, a DRL, a VI, a tandem DRL, and a tandem VI schedule in the multiple schedules groups, and a VR 8 schedule in the single schedule group. Once again, the functionality of these reinforcement contingencies depended on the extent to which they were useful to gain points under the FI 15 schedule. The results showed that overall, all groups failed to adapt to the task-contingencies during the last two minutes of the FI 15 schedule and that this was more prominent in the single schedule group, followed by the FR, the BOTH, and the DRL schedule groups, in that order.

**Studies included to answer Research Question 2.** Of those studies that met our inclusion criteria, only Baruch et al. (2007) examined whether psychological suffering moderated the RBIE. Specifically, Baruch et al. examined whether different types of instructions (plys and tracks) and the presence or absence of sub-clinical symptoms of depression differentially impacted adaption to task-contingency changes. Non-depressed (*n* = 14) and depressed (*n* = 15) undergraduate students were randomly given a ply or track which initially correctly described the task contingencies in a matching-to-sample (MTS) task, but in a subsequent phase were in contrast with these contingencies. The results revealed that both groups showed difficulties adapting to the new task-contingencies. However, relative to the non-depressed group, the depressed group adapted quicker to this change. No differences were observed as a function of the plys or tracks these groups received.

1. Broadly speaking, a ply specifies consequences delivered by the rule-giver for compliance with the rule (e.g., “I will give you money if you follow my [i.e., the experimenter] instructions”), while a track describes consequences that occur naturally when following the rule (e.g., “I will feel less pain if I take a pain-killer”). See Kissi et al. (2017) for more information on plys and tracks. [↑](#footnote-ref-1)
2. This number is based on an educated guess, given that the exact number of participants within each experimental group was not provided. It was merely stated that subjects were randomly assigned to one of the two experimental groups. [↑](#footnote-ref-2)
3. See Footnote 5. [↑](#footnote-ref-3)
