# Supplementary material for: The rule-based insensitivity effect: a systematic review
Source: PeerJ. 2020 Jul 23;8:e9496. doi: 10.7717/peerj.9496 (PMC7382939; doi:10.7717/peerj.9496)
Supplement: Supplemental Information 3 [file peerj-08-9496-s003.docx]

| **Studies used to answer Research Question 1 (“Is there evidence for the rule-based insensitivity effect in adults?”).** | | | | | | |  | |  |
| --- | --- | --- | --- | --- | --- | --- | --- | --- | --- |
|  | Non-random sequence generation  **(Selection Bias)** | Allocation revelation  **(Selection Bias)** | Prior testing  **(Selection**  **Bias)** | Misclassification of participants to experimental groups  **(Selection**  **Bias)** | Incomplete outcome data  **(Exclusion Bias)** | Selective reporting of outcomes  **(Reporting Bias)** | | Invalid and unreliable  outcome assessment methods  **(Detection Bias)** | |
| Cerutti  (1991) | ? | ? | ? | NA | ? | - | | - | |
| Cerutti  (1994) | ? | ? | ? | NA | ? | - | | - | |
| Dixon et al. (2000) | ? | ? | ? | + | ? | - | | - | |
| Haas and Hayes  (2006) | ? | ? | ? | + | ? | - | | - | |
| Harte et al. (2017 - Experiment 1) | ? | ? | ? | NA | ? | - | | - | |

| Harte et al.  (2017 - Experiment 2) | ? | ? | ? | + | ? | - | - |
| --- | --- | --- | --- | --- | --- | --- | --- |
| Hayes et al.  (1986) | ? | ? | ? | + | ? | - | - |
| Kissi et al.  (2018) | - | ? | ? | + | ? | - | - |
| Kudadjie-Gyamfi and Rachlin  (2002) | ? | ? | ? | + | ? | - | - |
| LeFrancois et al.  (1988) | ? | ? | ? | + | ? | - | - |
| Monestès et al.  (2017) | ? | ? | ? | + | ? | - | - |
| Monestès et al.  (2014) | ? | ? | ? | + | ? | - | - |
| Otto et al.  (1999 - Experiment 1) | ? | ? | ? | NA | ? | - | - |
| Otto et al.  (1999 - Experiment 2) | ? | ? | ? | NA | ? | - | - |

| Shimoff et al.  (1981) | ? | ? | ? | + | ? | - | - |
| --- | --- | --- | --- | --- | --- | --- | --- |
| Souza et al.  (2012) | ? | ? | ? | + | ? | - | - |
| Svartdal  (1989) | ? | ? | ? | NA | ? | - | - |
| Svartdal  (1995 - Experiment 2) | ? | ? | ? | NA | - | - | - |
| Torgrud et al.  (2006 - Experiment 1) | ? | ? | ? | NA | ? | - | - |
| Torgrud et al.  (2006 - Experiment 2) | ? | ? | ? | NA | ? | - | - |

|  | Inadequate outcome assessments  **(Detection Bias)** | Inadequateness of the method used to determine sample size  **(Detection Bias)** | Inappropriateness of analytic methods  **(Detection**  **Bias)** | Non-standardization of the experimental context  **(Performance Bias)** | Information about the study objectives  (**Performance bias**) | Non-Blinding of participants and personnel  (**Performance bias**) |  |
| --- | --- | --- | --- | --- | --- | --- | --- |
| Cerutti  (1991) | - | ? | ? | - | ? | ? |  |
| Cerutti  (1994) | - | ? | ? | - | ? | ? |  |
| Dixon et al.  (2000) | - | ? | ? | - | ? | ? |  |
| Haas and Hayes  (2006) | - | ? | ? | - | ? | ? |  |
| Harte et al.  (2017 - Experiment 1) | - | ? | ? | - | ? | ? |  |
| Harte et al.  (2017 - Experiment 2) | - | ? | ? | - | ? | ? |  |
| Hayes et al. (1986) | - | ? | ? | - | ? | ? |  |
| Kissi et al.  (2018) | - | ? | ? | - | ? | ? |  |

| Kudadjie-Gyamfi and Rachlin  (2002) | - | ? | ? | - | ? | ? |  |
| --- | --- | --- | --- | --- | --- | --- | --- |
| LeFrancois et al.  (1988) | - | ? | ? | - | ? | ? |  |
| Monestès et al.  (2017) | - | ? | ? | - | ? | ? |  |
| Monestès et al.  (2014) | - | ? | ? | - | ? | ? |  |
| Otto et al.  (1999 - Experiment 1) | - | ? | ? | - | ? | ? |  |
| Otto et al.  (1999 - Experiment 2) | - | ? | ? | - | ? | ? |  |
| Shimoff et al.  (1981) | - | ? | ? | - | ? | ? |  |
| Souza et al.  (2012) | - | ? | ? | - | ? | ? |  |
| Svartdal (1989) | - | ? | ? | - | ? | ? |  |

| Svartdal  (1995 - Experiment 2) | - | ? | ? | - | ? | ? |  |
| --- | --- | --- | --- | --- | --- | --- | --- |
| Torgrud et al.  (2006 - Experiment 1) | - | ? | ? | - | ? | ? |  |
| Torgrud et al.  (2006 - Experiment 2) | - | ? | ? | - | ? | ? |  |

**Note.** ‘+’, ‘-‘, and ‘?’ refer to high, low, and unclear risk of bias for a particular domain, respectively. NA means that the domain was not applicable.
