# Supplementary material for: The rule-based insensitivity effect: a systematic review
Source: PeerJ. 2020 Jul 23;8:e9496. doi: 10.7717/peerj.9496 (PMC7382939; doi:10.7717/peerj.9496)
Supplement: Supplemental Information 4 [file peerj-08-9496-s004.docx]

| **Studies used to answer Research Question 2 (“Do adults suffering from psychological problems display a larger RBIE compared to their non-clinical counterparts?”).** | | | | | | | |
| --- | --- | --- | --- | --- | --- | --- | --- |
|  | Non-random sequence generation  **(Selection Bias)** | Allocation revelation  **(Selection Bias)** | Prior testing  **(Selection**  **Bias)** | Misclassification of participants to experimental groups  **(Selection**  **Bias)** | Incomplete outcome data  **(Exclusion Bias)** | Selective reporting of outcomes  **(Reporting Bias)** | Invalid and unreliable  outcome assessment methods  **(Detection Bias)** |
| Baruch et al.  (2007) | ? | ? | ? | NA | ? | - | - |
|  | Inadequate outcome assessments  **(Detection Bias)** | Inadequateness of the method used to determine sample size  **(Detection Bias)** | Inappropriateness of analytic methods  **(Detection**  **Bias)** | Non-standardization of the experimental context  **(Performance Bias)** | Information about the study objectives  (**Performance bias**) | Non-Blinding of participants and personnel  (**Performance bias**) |  |
| Baruch et al.  (2007) | - | ? | ? | - | ? | ? |  |

**Note.** ‘+’, ‘-‘, and ‘?’ refer to high, low, and unclear risk of bias for a particular domain, respectively. NA means that the domain was not applicable
