# Supplementary material for: The rule-based insensitivity effect: a systematic review
Source: PeerJ. 2020 Jul 23;8:e9496. doi: 10.7717/peerj.9496 (PMC7382939; doi:10.7717/peerj.9496)
Supplement: Supplemental Information 5 [file peerj-08-9496-s005.docx]

1. **The rationale for conducting the systematic review**

Despite the attention that rules and the rule-based insensitivity effect (RBIE) have received and the presumed importance of this effect for our understanding of human behavior in general and psychological suffering in particular, there is currently no systematic review available of the experimental work examining this effect. As such, this systematic review aimed to begin to fill in this gap by systematically reviewing the RBIE literature to examine if: (1) there is sufficient empirical support for this effect in adults, and (2) adults suffering from psychological problems display larger levels of this effect compared to those that do not suffer from these problems. We also investigated how (3) different operationalizations of the RBIE, and (4) the external validity and risks of bias of the experimental work investigating this effect, might influence the conclusions that can be drawn from the current systematic review.

The rationale for conducting the systematic review can be found on pages 3-4 (see the introduction).

1. **The contribution that the systematic review makes to knowledge in light of previously published related reports, including other meta-analyses and systematic reviews**

The current systematic review is the first to systematically synthesize the available experimental work on the RBIE. The results revealed that despite the widespread appeal that the RBIE has enjoyed, at present, only preliminary evidence exists for the idea that adults demonstrate the RBIE and no evidence is available to assume that psychological problems exacerbate the RBIE in adults.

See the last paragraph of the introduction (page 4), and the discussion and conclusion sections for information about the contribution of the systematic review.
